# Twitter social mobility data reveal demographic variations in social distancing practices during the COVID-19 pandemic

**DOI:** 10.1038/s41598-024-51555-0

**Published:** 2024-01-12

**Authors:** Paiheng Xu, David A. Broniatowski, Mark Dredze

**Affiliations:** 1 Department of Computer Science, University of Maryland, College Park, MD 20742 USA; 2 Department of Engineering Management and Systems Engineering, The George Washington University, Washington, DC 20052 USA; 3 Department of Computer Science, Johns Hopkins University, Baltimore, MD 21218 USA

**Keywords:** Epidemiology, Computational science

## Abstract

The COVID-19 pandemic demonstrated the importance of social distancing practices to stem the spread of the virus. However, compliance with public health guidelines was mixed. Understanding what factors are associated with differences in compliance can improve public health messaging since messages could be targeted and tailored to different population segments. We utilize Twitter data on social mobility during COVID-19 to reveal which populations practiced social distancing and what factors correlated with this practice. We analyze correlations between demographic and political affiliation with reductions in physical mobility measured by public geolocation tweets. We find significant differences in mobility reduction between these groups in the United States. We observe that males, Asian and Latinx individuals, older individuals, Democrats, and people from higher population density states exhibited larger reductions in movement. Furthermore, our study also unveils meaningful insights into the interactions between different groups. We hope these findings will provide evidence to support public health policy-making.

## Introduction

Social distancing and isolation are among the most effective methods to mitigate the spread of viral outbreaks. Especially early in a pandemic when little is known and other interventions are unavailable, preventing physical proximity between susceptible individuals can reduce virus transmissions and protect vulnerable populations^1–3^. At the start of the COVID-19 pandemic in the United States, public health officials requested that the public avoid large gatherings and limit contact with others as part of a social distancing initiative^4^. Subsequent research demonstrated the effectiveness of these social distancing guidelines^5–7^. However, mixed compliance with these recommendations limited their effectiveness. A number of factors may negatively affect social distancing, including financial factors, the housing environment, and distrust of public officials^8–11^. Furthermore, experiences during the pandemic have motivated calls for research into models that include the likelihood of compliance related to social factors in pandemic forecasting models^12–15^. Understanding who adheres to social distancing and what factors influence these practices may be critical to ensuring the effectiveness of these practices.

One way we can measure compliance is through online mobility data, a quantitative measure of travel patterns^16^. Near real-time measures of mobility from, for example, GPS-enabled mobile phones, offer a massive, detailed indicator of mobility patterns, and have thus been used during the COVID-19 pandemic^17–19^. In contrast, traditional survey data can be time-consuming to collect and suffers from response bias^20^. However, a drawback of these data compared to surveys is that they are only available in aggregated form, and thus cannot associate mobility with other factors. Critically, we cannot answer who has reduced their mobility, and what factors are related to this decision.

Following research that uses social media data for public health^21–25^, work during the COVID-19 pandemic (and in previous epidemics^26^) has turned to Twitter as an alternative source of mobility data^27,28^. At the time the study was conducted, public tweets could be collected on an ongoing basis in real-time through Twitter’s publicly available Application Programming Interface (API) for free. Twitter changed such access to paid service in 2023. Important for our purposes, Twitter allows for geotagging tweets, which then includes location information in the tweet metadata. Additionally, numerous studies have explored automatic Twitter geolocation^29–32^, including work on patterns and trends in Twitter geotagged data^33^. In this study, we rely on public Twitter posts that contain user-provided location data.

### Social distancing and demographics

 An advantage to Twitter data is that we can observe other information about the user who posted the tweet, which allows us to study how mobility changes correlate with individual characteristics related to health behaviors, such as age^34–36^, income^37,38^, race^39^, and political affiliation^9,10,40^. For example, partisanship is more strongly associated with physical distancing than other factors in the United States^10^. Prior work has demonstrated how to infer relevant characteristics from Twitter data^41–44^, including gender^45–47^, race/ethnicity^48,49^, age^50^, and political affiliation^51,52^. Demographic factors (gender, age, race/ethnicity, political party) help predict intent to adhere to social distancing but are relatively poor predictors compared with individual attitudes and media diets^15^. Similarly, cellular mobility data at the county level can be used to reveal patterns in social distancing correlated with partisanship, media consumption, and racial and ethnic composition, as well as to measure the effectiveness of interventions that promote social distancing^53^. Twitter data provides a complement to these data sources, allowing for confirmation of population-level trends as well as more fine-grained analysis.

### Mask usage

 Face masks have been another critical intervention adopted during the pandemic. The adoption of mask requirements and compliance have varied dramatically by location and jurisdiction^54^. Chernozhukov et al. causally evaluate the impact of various policies of U.S. states and social distancing behavior measured by Google Mobility Reports on the spread of COVID-19 cases, demonstrating that nationally mandating face masks for employees early in the pandemic could have largely reduced the growth rate of cases and deaths^55^. Similarly, Eikenberry et al. studied mask effectiveness in New York State and Washington State^56^. Others have concluded that mandatory mask policies increase mask usage as compared to voluntary policies^57^.

### Contribution

**Figure 1:**
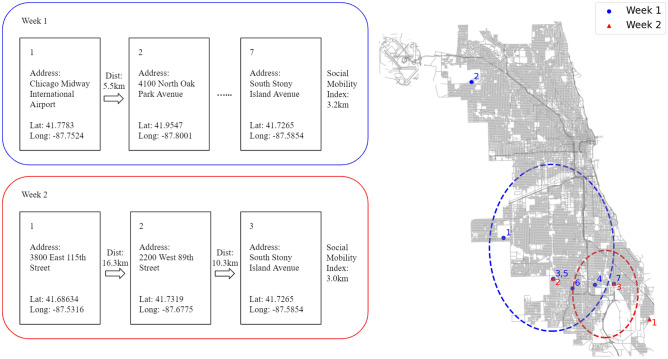
Visualization of Twitter Social Mobility Index. A Twitter user’s trajectories for Week 1 (blue) and Week 2 (red) are shown on the map of Chicago while the detailed location information is shown on the left. Each point on the map represents a coordinate derived from the user’s check-in Tweet. The number by the point is the order in which the tweet occurred. Each circle is centered at the centroid location for that week, and the radius of the gyration shows the distance traveled. The Twitter Social Mobility index is computed by the standard deviation of distance traveled across locations within each week.

 We use data from the Twitter Social Mobility Index^27^ (Fig. 1) to study how demographic characteristics and political affiliation correlate with changes in mobility patterns, revealing insights into social distancing practices. We use demographic inference techniques, combined with user-level mobility data, to examine how different groups responded to the COVID-19 pandemic in the United States. We describe in detail the Twitter Social Mobility Index data and demographic characteristics in the Methods. Our findings can inform public health messaging and identify communities at higher risk from the virus.

## Results

Overall we found the following groups in the United States exhibited larger reductions in mobility as compared to counterpart groups based on analysis of variance (ANOVA) tests: males, Asian and Latinx individuals, older individuals, Democrats, and people from higher population density states. The conclusions on characteristics with multiple groups, i.e. race/ethnicity and political affiliation, are supported by Tukey’s test (see Supplementary Tables S6 and S7 online). In the following section, we explain each of these findings in detail and ground them in the literature.

### Political affiliation

#### Hypothesis

 (1) Democrats reduced mobility more than Republicans, and (2) political affiliation acts as the main effect in interactions with other variables such as population density, race/ethnicity, and age.

#### Background

 In the United States, political affiliation has been identified as a significant factor in determining COVID-19 behaviors. After a government order, residents in Democratic counties are more likely to stay home relative to those in Republican counties, based on geolocation data from SafeGraph^9^. Similar results were found using smartphone location data^10,40^. Additionally, population density confounds political affiliation as Democrats are more likely to live in dense, urban areas and thus be subject to stricter policies^40^. Nevertheless, Allcott et al. and Gollwitzer et al. controlled for population density and still found that people from areas with more Democrats reduced mobility more^10,40^. Gollwitzer et al also found partisanship is more strongly associated with physical distancing than numerous other factors, including counties’ COVID-19 cases, population density, median income, and racial and age demographics^10^.

Another breakdown can examine support for US President Donald Trump, who was president during the start of the COVID-19 pandemic. Recent work suggests Trump supporters are less likely to accept COVID-19 vaccines^58^. Painter et al. studied the effect of Trump’s initial message which downplayed the severity of the coronavirus pandemic^9^ as the press suggested that Republicans may not take social distancing seriously. They found Democratic counties with Republican governors have lower responses relative to the aligned Democratic counties and there were no significant differences among Republican counties.

#### Result

 From Table 1 and Supplementary Table S7, we observe that Democrats have a significantly larger mobility reduction than Republicans. Regarding the interactions between political affiliation and other variables, we follow the interaction analysis from our major ANOVA test in Supplementary Table S9 and the post-hoc analysis in Supplementary Table S8. We find that Democrats have a larger mobility reduction than Republicans, regardless of whether they are in high or low-population-density states. Furthermore, political affiliation has a larger impact than gender. All Democrats have larger mobility reductions regardless of gender. We also observe a significant interaction with three variables, i.e. gender, age, and political affiliation. After ignoring groups with unknown political affiliations, we find 12/28 significant comparisons. 9/12 out of these comparisons are between Democrat groups and Republican groups. For each of these comparisons, the Democrat group has a significantly larger mobility reduction compared to the Republican group, demonstrating the strong impact of political affiliation. Finally, Supplementary Table S12 implies that users who follow Trump on Twitter have a larger mobility reduction, although the difference is small.

### Race/ethnicity

#### Hypothesis

 we believe that (1) Black and Latinx groups show smaller mobility reduction while Asian group shows the opposite. As many studies control for population density^10,40^, we expect (2) there are significant differences in the interaction of race/ethnicity and population density.

#### Background

 Gollwitzer et al. found counties with higher Black or Latinx populations were less likely to reduce general mobility and non-essential visits, while Asian populations were in favor of these reductions^10^. Census data combined with COVID-19 tests show that individuals from poor and immigrant neighborhoods and areas with predominantly Black populations in New York City are more likely to test positive^39^.

#### Result

 The results partly support our hypothesis. From Supplementary Tables S6 and S8, we find that Asian and Latinx groups have a larger mobility reduction compared to Black and White groups. Furthermore, state population density has little impact when interacting with race/ethnicity as shown in Supplementary Table S8. The significant differences between the groups mostly follow the trends for race. For all race/ethnicity groups, there is no significant difference between people from high and low-population-density states in this interaction.

### Age

#### Hypothesis

 (1) Older individuals have a larger mobility reduction. (2) However, we expect significant interactions between multiple demographics, namely between age and race/ethnicity.

#### Background

 Previous studies found people born before 1965 are more likely to practice social distancing than people born between 1981 and 1996 using an online survey^36^. Counties with higher median ages showed larger reductions in movement^10^.

#### Result

 Table 1 confirms our hypothesis on older individuals. The results are more mixed for the age and race interaction in Supplementary Table S8. Older people reduce their mobility more when compared with other younger groups regardless of race/ethnicity except for the comparisons involving Black people over 30. Black individuals have the smallest mobility reduction among all combinations for people over 30 in this interaction. They have a significantly smaller reduction when compared to Asian people below 30, and have no significant differences when compared to Latinx and White people below 30. For each of the four race/ethnicity groups included in our study, older people have significantly larger mobility reductions, showing age is the main effect when interacted with race/ethnicity.

### Gender

#### Background

 Allcott et al. found that there were no statistically significant differences in social distancing behaviors by gender^40^. Similarly, we do not expect to detect any significant difference between males and females.

#### Result

 Looking at the interaction between gender and age from Supplementary Table S8, we find that age has a larger impact on mobility; younger people have a smaller reduction when compared with other older groups regardless of gender. The differences between gender and other variables only exist for certain groups. For males and females in the same age range, we observe that males have a significantly larger mobility reduction ($11.3\%$, $p<0.001$) than females do when over 30. Male Republicans have $18.5\%$ ($p<0.05$) larger mobility reduction than female Republicans, but we cannot conclude a significant difference between Democrat males and females.

### Content analysis

We also include Twitter content characteristics, i.e. mentioning of COVID-19 and social-distancing hashtags, in separate analyses to validate our method, shown in Supplementary Tables S10 and S11. People who tweeted COVID-19 related hashtags have a larger reduction in mobility. We cannot conclude the same for people tweeting about social-distancing hashtags due to the relatively small sample size for the five-way ANOVA analysis. However, for the one-way ANOVA that includes all the above-mentioned characteristics, Supplementary Table S13 confirms the intuitions that people tweeted COVID-19 and social distancing related hashtags reduce more mobility, and it also supports our previous conclusions on the other characteristics.

### Mobility index distribution

**Figure 2:**
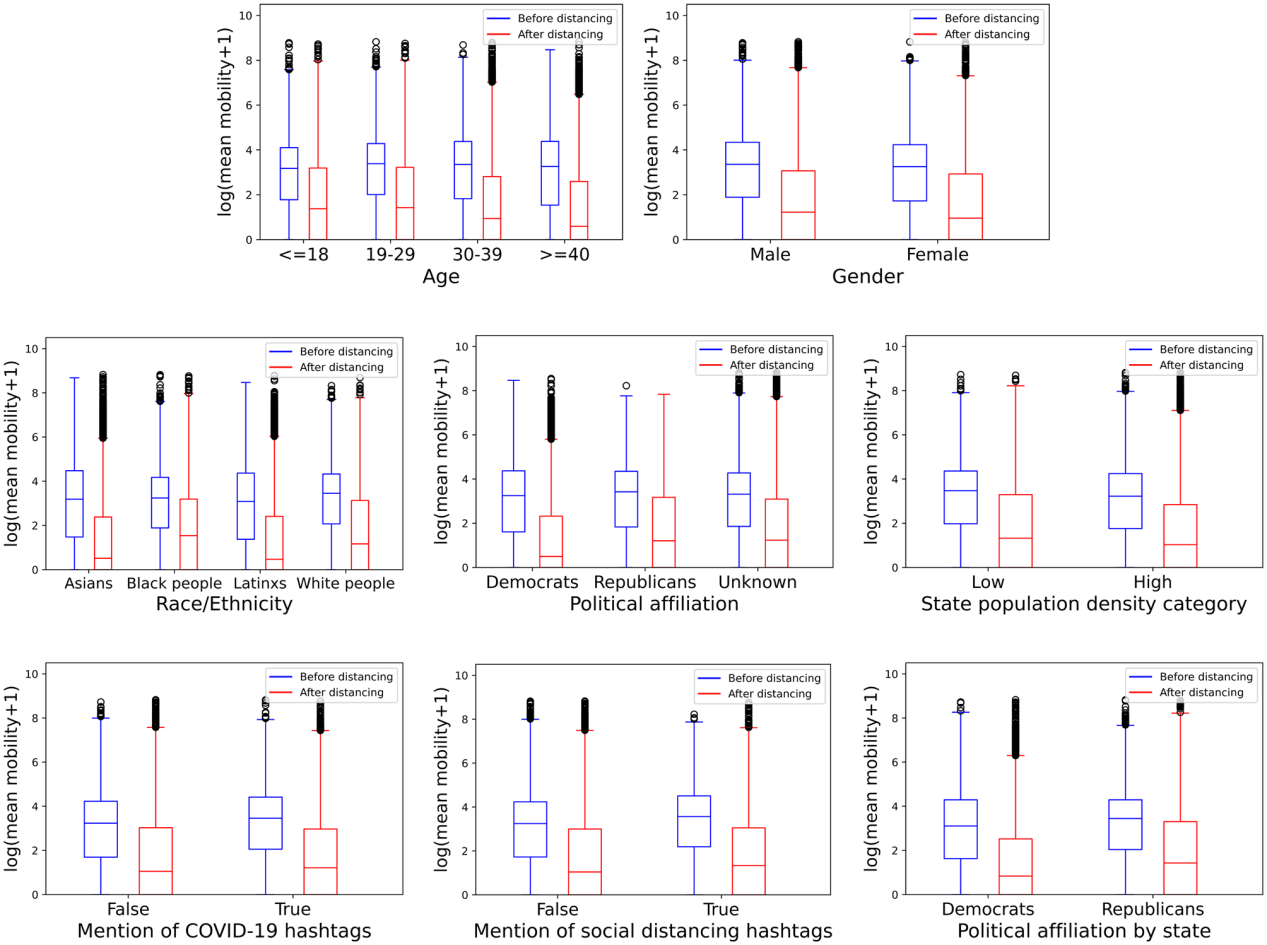
Mobility index distributions of each characteristic before (blue) and after (red) the start of the COVID-19 pandemic.

Figure 2 shows changes in mobility from before and after the start of social distancing for each characteristic in our dataset. Each sub-group displays a reduction in mobility, consistent with the ANOVA tests. We can also see differences by attributes: we observe larger reductions for older individuals, people from high-density states, and Democrats. We note that high-density areas may have seen a larger drop because individuals can find services in a smaller geographic area.

### Regression analysis

Beyond reductions correlated with different demographic groups, we hypothesize that trust in government and perceived risk are associated with social distancing behavior. Our regression analysis, described in the "Methods" Section with results in Supplementary Tables S15, S16 and S17, shows that trust in the government predicts social distancing behavior even after controlling for perceived risk and population density at the state level.

**Table 1:** Summary statistics of ANOVA tests and mean mobility differences. F statistic, DF (degrees of freedom), and p value are shown for each variable.

Variable	F statistic	DF	P-value	Group	Mean mobility difference	Sample size
Gender	6.80	1.0	$9.13\times 10^{-03}$	Female	32.48	194414
				Male	36.40	258625
Race/ethnicity	24.50	3.0	$7.60\times 10^{-16}$	White people	35.65	184654
				Asians	47.11	40193
				Latinxs	42.70	81509
				Black people	25.72	146683
Age	22.85	1.0	$1.75\times 10^{-06}$	$< 30$	28.40	245202
				$\ge 30$	42.18	207837
Political affiliation	8.05	2.0	$3.18\times 10^{-04}$	Unknown	32.70	363927
				Democrats	45.53	63955
				Republicans	36.44	25157
State population density category	10.50	1.0	$1.19\times 10^{-03}$	High	35.69	295719
				Low	32.90	157320

## Discussion

Our analysis concludes that male users, Asian and Latinx users, older users, Democrats, and people from higher population density states showed larger reductions in mobility. For race/ethnicity, the observations of Asian and Black populations confirm previous work^10^. However, in our analysis, Latinx people are more likely to reduce movement. This might be due to the relatively poor performance of the race/ethnicity inference model on Latinxs (See Supplementary Tables S3 and S4 online). We are surprised to observe a significant gender difference which might be caused by the imbalanced age distribution in our dataset, i.e. the sample sizes between males and females are close when under 30 while there are many more male users relative to females over 30. In general, a consistent picture emerges across multiple data sources and analyses that indicates that some groups practice less social distancing, and thus may be at a higher risk of infection.

Moreover, our study provides meaningful insights into the interactions between different groups. For example, political affiliation shows a stronger impact when interacting with gender and age, which is similar to the findings from smartphone data^10^. Age is another strong factor since older people have a larger mobility reduction regardless of gender, as we expected. However, when it interacts with race/ethnicity, we find an exception for Black people as older Black people show smaller mobility reduction than some of the younger people from other race/ethnicity groups.

We emphasize that our results do not indicate willingness or attitudes towards social distancing or mobility reduction of certain groups. We are measuring actual behaviors, which may not align with attitudes for a variety of reasons. For example, individuals may agree with the importance of social distancing but may still need to travel for economic reasons. Weill et al. showed wealthy areas reduced mobility more during the pandemic^37^. Population density has less impact when interacting with other demographics, which contradicts our hypothesis, which may be due to the fact that our population density labels are at the state, and not individual, level. There are dense, urban areas in states with low population density and vice versa.

A major advantage of Twitter data over other mobility data sources is the ability to link to comments and other behaviors of the users. We utilize this ability to show that increased trust in government correlates with greater mobility reductions across age and race/ethnicity. Public health communication strategies should consider how to best reach this at-risk group.

Our results demonstrate the utility of data from the Twitter Social Mobility Index. Future work on social distancing and health behaviors during epidemics can utilize similar public Twitter data to measure the effectiveness of public health policies. Still, future analyses must recognize the limitations of this data source, which we discuss in the "Methods" section. Critically, our analysis considers individual characteristics rather than a holistic analysis that may illuminate other issues. For example, Democrats may be more likely to reduce their mobility because they live in dense urban areas. Additional unavailable variables may form critical parts of the story, but we lack this information. For example, we do not have access to socioeconomic information, but these factors may be confounding variables that explain why some groups have smaller reductions in mobility. Additionally, following other studies, we measure social distancing as reflected through social mobility. While there is significant evidence to support this association, the correlation is not perfect. Individuals may have increased mobility but stayed away from others (e.g. travel to rural areas), decreased mobility but increased social interactions (in-person gatherings in their neighborhood), or increased mobility but adopted other precautions (6 feet distancing, masking.) We cannot measure the difference between mobility and other precautions using our data. Furthermore, the dramatic social changes from the pandemic may have influenced how users geotagged their data, perhaps leading to changes in the social mobility measure. While the large drop in social mobility at the start of the pandemic strongly suggests a causal link between the pandemic and mobility, we cannot rule out other unobserved factors.

Overall, our analysis illustrates the value of geolocated Twitter data in understanding public health behaviors during a pandemic.

## Methods

### Data collection

We use data collected as part of the Twitter Social Mobility Index Project^59^ This data includes public geotagged tweets from the United States from January 1, 2019 to June 21, 2020. The index is computed by aggregating geotagged data for a user and measuring the standard deviation across locations within each week. A high standard deviation in a week means high mobility. Changes in mobility behavior are measured over time by comparing mean mobility week to week. See Xu et al. for more details on data and computing mobility^27^.

We select March 16, 2020 as the start of social distancing in the United States, since the national “Slow the Spread” guidelines announced on that date had the largest effect on mobility^27^. Furthermore, Badr et al. showed mobility changes in many US counties following this announcement, even before individual state-level policies were implemented^19^. We compare the time period before (January 1, 2019 - March 15, 2020) and after (March 16, 2020 - June 21, 2020) this date as “before” and “after” the start of the pandemic. Our analysis relies on inferring demographics and analyzing the content of tweets. Therefore, we download the 3,200 most recent tweets for each of the 505,589 Twitter users in the collection who are present both before and after the start of the pandemic. We exclude 51,447 users identified as organizations by either of the existing individual vs. organizational account systems^50,60^, leading to a total of 454,142 Twitter users. Our user-level dataset contains one entry for each user, including the mobility index, number of weekly geotagged tweets, and mean mobility index before and after the start of the pandemic. On average, the users in the dataset have 14.25 geotagged tweets weekly.

We augment each user in the dataset with demographic information as follows.

#### Location

 The user’s home city and state are computed from the centroid of all of their geotagged tweets. In our analysis, we use the home location to categorize a user as living in a high or low population density state, with a threshold set as the median US state population density^61^.

#### Age and gender

 Age and gender are inferred using M3^50^, which uses both image (profile image) and text (name, username, user self-description) features. We use the text-based model when the profile image is unavailable. For gender, the full model achieves 0.918 macro-F1 and the text-only model 0.907 when evaluated on heuristically-labeled self-report data. For age, the full model achieves a 0.522 macro-F1 score. M3 produces age categories of 18 and under, 19-29, 30-39, and over 39. In our ANOVA analysis, we simplify this to be over/under 30. The macro-F1 score for simplified categories is 0.700.

#### Race/ethnicity

 We include categorical race/ethnicity based on the model using DistilBERT^62^ to embed the latest 200 tweets of each user into a fixed-length representation, which is then passed through a logistic regression with l2 regularization^49^. The model achieves 0.513 macro-F1 and $52.6\%$ accuracy on a balanced dataset of self-reported race/ethnicity labels^48^. Both the model and evaluation dataset provide the following race/ethnicity labels: White people, Black people, Asians, and Latinxs. We note that there are other racial groups in the US. The current categorizations are limited by the inference tool and evaluation data available. We provide the confusion matrices for age, gender, and race/ethnicity inference models in Supplementary Tables S1, S2, S3 and S4.

#### Political affiliation

 We identify political affiliation in the United States (Democrat or Republican) using a strategy similar to Preotiuc-Pietro et al^51^. We use three methods and include each in the dataset. (1) A user is assigned a label if they follow a member of congressional leadership from *either* the Democrats – Nancy Pelosi (@SpeakerPelosi) or Chuck Schumer (@SenSchumer) – or the Republicans – Kevin McCarthy (@GOPLeader) or Mitch McConnell (@senatemajldr). Otherwise, they are assigned the label unknown. (2) We apply the same approach but consider all members of Congress in 2020^63^. We use the labels produced by this approach in our ANOVA analysis. (3) We also assign political affiliation labels based on the home state’s vote in the 2016 US presidential election^64^. We excluded former President Trump’s account from this method since it is very popular and widely followed. Instead, we indicate if a user follows the former US President as a separate field, which may be useful as some studies have found that Trump supporters are less likely to accept a future COVID-19 vaccine^58^.

We include two characteristics that reflect tweeted content.

#### COVID-19 hashtags

 We indicate if this user tweeted or retweeted a COVID-19 hashtag in their most recent 3200 tweets. We collected hashtag usage from $81.1\%$ of all the active users since 2020 March in^27^, which is 1,103,749 users. We then manually identified COVID-19 hashtags by examining the 427 most popular hashtags whose total usage is above 30,000 tweets.

#### Social-distancing hashtags

 We repeat the same process to identify social distancing hashtags. The hashtags for COVID-19 and social distancing are listed in Supplementary Table S5.

### ANOVA tests

We run ANOVA tests to determine whether the differences in mobility reduction among the demographic groups are significant. For all ANOVA tests in this study, we use the difference in mobility (reduction) as the dependent variable. We select age, gender, race/ethnicity, and political affiliation considering all current members of Congress, and state population density as independent variables for our major ANOVA test. We show the summary statistics of these five variables in Table 1, and significant interactions and mean mobility difference in Supplementary Table S9. We run three other separate ANOVA tests where we replace political affiliation with indicators of whether a user mentions COVID-19, or social distancing-related hashtags, and whether a user follows former U.S. president Trump. The summary statistics are shown in Supplementary Tables S10, S11 and S12 respectively. For the major ANOVA test, we also conduct post-hoc analysis by running a pairwise t-test to compare the groups in each significant interaction cell. We use Bonferroni correction to reduce the likelihood of committing Type I Error. The mean mobility difference and corrected p-values for post-hoc analysis are shown in Supplementary Table S8.

### Regression analysis

We run a linear regression to test our hypothesis that social distancing behavior is associated with trust in government and perceived risk. We further augment each user in our dataset with trust-in-government measures based on age and race/ethnicity, and perceived risk based on state. We obtain trust measures for race/ethnicity and age from the Pew Research Center. We used the datapoint on 8/2/2020 which covers 4 race/ethnicity groups considered in our analysis. Original age trust measure data covers 5 generations. We used a weighted average by population from the US Census Bureau to generate trust measures for those below (born after 1981) and above 30 (born before 1980). The generated trust measures are 19.0 and 20.3 accordingly. We measure perceived risk using the cumulative confirmed cases for each state on 6/21/2020 which is the end of our mobility dataset. We note that the trust-in-government measures are aggregated numbers and we do not include them in the proposed user-level dataset. We use these aggregated numbers that link to users’ race and age groups to test the related hypothesis. We leave building a reliable trust-in-government inference model on Twitter for future works.

We first include perceived risk and categorical demographics predictors, i.e. age, gender, political affiliation, and race/ethnicity, as independent variables. We then replace age and race with trust in government measures, controlling for perceived risk. To tease apart rural from trust-related variables, we also control for the state population density. We use the exact density values in this analysis. Considering that the dependent variable, users’ mobility reduction, is not normally distributed as shown in Supplementary Fig. S1, we split the users into two groups based on whether their mobility reduction is over or below 0. Users with 0 mobility both before and after March 16, 2020 are removed from the analysis. The log-transformed mobility reduction distribution plots for these two groups are shown in Supplementary Fig. S2. We run a linear regression on users who have mobility reduction over 0 with the above-mentioned experiments. We then log-transform the reduction with $log(x+1)$ and repeat the linear regression analysis. Finally, we run a logistic regression comparing users who have a mobility reduction over 0 to those who have a reduction of 0 or less in the same settings. When using dummy encoding for categorical variables, the reference group is the largest one except for political affiliation’s reference group is Democrats where the largest group is unknown. Min-max normalization is applied for each numerical dependent variable. The results are in Supplementary Tables S15, S16 and S17.

### Data limitation

A responsible analysis must contextualize our results with the known, and potentially unknown, limitations of our data and methods. We enumerate some of these issues.

Twitter is a biased source of data on a population. It reflects a non-random sample of the underlying population, and users choose to share different types of information and use the platform in different ways^65^. For example, different demographic groups do not use geotagging with the same prevalence^66^. Demographics like age and gender introduce bias that interacts with geographic inference and how geotagging may be used on Twitter^32^. Similar demographic bias is also found in mobility data from cell phones, i.e. older and non-White users are less likely to be captured^67^. While Twitter has yielded numerous insights into population health^21^, we must remain cautious about this source of bias as we explore each new issue. We note that at the time of data collection for this study, Twitter provided free access to its data for academic purposes. However, the recent policy change regarding access to Twitter data has introduced greater challenges in utilizing it as a data source for research.

Furthermore, our methods for inferring demographic information, including gender, age, and race/ethnicity are far from perfect. We report the accuracies of our selected systems in the body of the paper. Beyond raw accuracy, these systems all have biases in how they make demographic inference decisions. They mostly capture perceived demographics, which may not be consistent with an individual’s self-identified demographics. Furthermore, prior work has shown that different demographic groups may use Twitter differently, a factor that is not captured by demographic inference systems or our own analysis^66^. The demographic inference models we use are limited in that they do not cover all groups within a demographic characteristic. For example, we combine race and ethnicity into 4 groups supported by the data and models but exclude other groups in the United States. Similarly, our gender models reflect only cisgender labels and exclude gender minorities. These limitations in data and available systems must be considered when drawing conclusions from our analysis.

Despite the massive size of our dataset, there are many gaps. We have only a few geotagged tweets from each user each week, and we do not have enough data to produce county-level analyses for most locations in the United States. Therefore, these results should be compared to those from other data sources, and further work should more fully explore specific conclusions of the analysis.

### Ethics

We must consider issues of ethics and privacy when mining social media data, even when it is data publicly posted online. There are different ethics and privacy issues to be considered when using Twitter data versus other mobility data, such as from mobile phone use. While mobile phone data are private and potentially very sensitive, they are not widely available, nor do they contain message content. In contrast, our Twitter data is (potentially) less sensitive and publicly available, but contains the text of messages. We must be sensitive to unintentional privacy violations that occur when analyzing aggregated data from a single user. More generally, when addressing issues related to health, attention to privacy is critical^68^.

In our work, we aggregated all mobility metrics to produce population-level analyses. None of our work considers the identity of individual users, and we removed identifiable user information from the distributed data aggregations. Furthermore, we caution others who pursue work similar to ours to consider privacy ramifications for users when collecting new data and conducting similar analyses. Finally, this research is conducted under an IRB-approved exemption under 45 CFR 46 category 4.

### Supplementary Information

Supplementary Information.

## Data Availability

Our data are public tweets containing user-provided geolocation information. To protect user privacy we remove all content and only use the information described in our analysis. The data that support the findings of this study are available from the corresponding author upon reasonable request.
