# Supplementary material for: Twitter social mobility data reveal demographic variations in social distancing practices during the COVID-19 pandemic
Source: Sci Rep. 2024 Jan 12;14:1165. doi: 10.1038/s41598-024-51555-0 (PMC10786940; doi:10.1038/s41598-024-51555-0)
Supplement: Supplementary file 1 — Supplementary Information. [file 41598_2024_51555_MOESM1_ESM.pdf]

Twitter Demographics Inference Models

This section lists the confusion matrices of Twitter demographics (age, gender, and race/ethnicity) inference model on a golden survey dataset[48]. Table S1, S2 and S3 are confusion matrices for age, gender, and race/ethnicity respectively, evaluated on the whole golden dataset retrieved in 2019. Considering the highly imbalanced distribution of race/ethnicity labels, we create a balanced dataset by subsampling. Table S4 is the confusion matrix evaluated on this balanced dataset.

	<30	>=30
<30	31.6	8.9
>=30	18.3	41.2

Supplementary Table S1. Confusion matrix for age inference model[50] on Twitter.

	female	male
female	58.9	7.6
male	2.5	31

Supplementary Table S2. Confusion matrix for gender inference model[50] on Twitter.

	White people	Black people	Latinxs	Asians
White people	42.9	11.4	11.3	15.8
Black people	1.7	5.5	0.8	1.4
Latinxs	1.9	1.1	1.6	1.3
Asians	0.7	0.3	0.2	2.0

Supplementary Table S3. Confusion matrix for race/ethnicity inference model[49] on Twitter.

	White people	Black people	Latinxs	Asians
White people	13.1	4.0	3.1	4.9
Black people	4.2	17.1	1.3	2.4
Latinxs	7.6	4.9	6.9	5.3
Asians	5.3	2.4	1.8	15.4

Supplementary Table S4. Confusion matrix for race/ethnicity inference model[49] on Twitter, evaluated on a balanced dataset.

COVID-19 and Social Distancing Related Hashtags

Topic	Hashtags
COVID-19	covid19, coronavirus, covid-19, covid_19, covid, coronaviruspandemic, covid2019
Social distancing	stayhome, socialdistancing, quarantine, quarantinelife, stayathome

Supplementary Table S5. Most popular hashtags identified that are related to COVID-19 and social distancing.

Tukey's Test

Group 1	Group 2	Mean mobility reduction difference	Adjusted p value
Asians	Black people	-21.8611	0.001
Asians	Latinx	-4.9045	0.001
Asians	White people	-11.9598	0.001
Black people	Latinx	16.9565	0.001
Black people	White people	9.9013	0.001
Laxinx	White people	-7.0552	0.001

Supplementary Table S6. Pairwise Tukey's test statistics for race/ethnicity.

Group 1	Group 2	Mean mobility reduction difference	Adjusted p value
unknown	Democrats	12.7481	0.001
unknown	Republicans	3.6928	0.001
Democrats	Republicans	-9.0553	0.001

Supplementary Table S7. Pairwise Tukey's test statistics for political affiliation.

Post-hoc Analysis

Interaction	Group 1	Group 2	Mean mobility difference		t-statistic	DF	Corrected p values
			Group 1	Group 2			
Gender× Age	female and < 30	female and ≥ 30	28.46	39.27	-17.01	194413	1.04×10^{-62}
	female and < 30	male and < 30	28.46	28.34	0.23	245201	1.00
	female and < 30	male and ≥ 30	28.46	43.72	-27.13	257692	1.17×10^{-159}
	female and ≥ 30	male and < 30	39.27	28.34	16.18	195345	1.15×10^{-56}
	female and ≥ 30	male and ≥ 30	39.27	43.72	-6.45	207836	1.62×10^{-8}
	male and < 30	male and ≥ 30	28.34	43.72	-26.23	258624	2.86×10^{-149}
Race/Ethnicity× Age	White and < 30	White and ≥ 30	30.83	41.74	-19.42	184653	8.90×10^{-82}
	White and < 30	Asian and < 30	30.83	36.50	-5.46	123861	6.92×10^{-6}
	White and < 30	Asian and ≥ 30	30.83	58.45	-25.93	122546	1.06×10^{-145}
	White and < 30	Laxinx and < 30	30.83	34.57	-4.18	127960	4.15×10^{-3}
	White and < 30	Laxinx and ≥ 30	30.83	46.26	-22	159763	5.80×10^{-105}
	White and < 30	Black and < 30	30.83	22.47	15.17	199594	8.26×10^{-50}
	White and < 30	Black and ≥ 30	30.83	31.97	-1.69	153303	1.00
	White and ≥ 30	Asian and < 30	41.74	36.50	4.59	102299	6.43×10^{-4}
	White and ≥ 30	Asian and ≥ 30	41.74	58.45	-14.26	100984	5.86×10^{-44}
	White and ≥ 30	Laxinx and < 30	41.74	34.57	7.29	106398	4.43×10^{-11}
	White and ≥ 30	Laxinx and ≥ 30	41.74	46.26	-5.84	138201	7.64×10^{-7}
	White and ≥ 30	Black and < 30	41.74	22.47	31.61	178032	1.37×10^{-216}
	White and ≥ 30	Black and ≥ 30	41.74	31.97	13.14	131741	2.76×10^{-37}
	Asian and < 30	Asian and ≥ 30	36.50	58.45	-10.3	40192	1.03×10^{-22}
	Asian and < 30	Laxinx and < 30	36.50	34.57	1.09	45606	1.00
	Asian and < 30	Laxinx and ≥ 30	36.50	46.26	-6.79	77409	1.60×10^{-9}
	Asian and < 30	Black and < 30	36.50	22.47	12.36	117240	6.18×10^{-33}
	Asian and < 30	Black and ≥ 30	36.50	31.97	3.34	70949	1.21×10^{-1}
	Asian and ≥ 30	Laxinx and < 30	58.45	34.57	13.21	44291	1.32×10^{-37}
	Asian and ≥ 30	Laxinx and ≥ 30	58.45	46.26	8.26	76094	2.12×10^{-14}
	Asian and ≥ 30	Black and < 30	58.45	22.47	30.89	115925	1.46×10^{-206}
	Asian and ≥ 30	Black and ≥ 30	58.45	31.97	19.07	69634	9.89×10^{-79}
	Laxinx and < 30	Laxinx and ≥ 30	34.57	46.26	-9.4	81508	8.23×10^{-19}
	Laxinx and < 30	Black and < 30	34.57	22.47	12.29	121339	1.48×10^{-32}
	Laxinx and < 30	Black and ≥ 30	34.57	31.97	2.25	75048	1.00
	Laxinx and ≥ 30	Black and < 30	46.26	22.47	31.37	153142	3.55×10^{-213}
	Laxinx and ≥ 30	Black and ≥ 30	46.26	31.97	15.3	106851	1.25×10^{-50}
	Black and < 30	Black and ≥ 30	22.47	31.97	-12.9	146682	6.46×10^{-36}
Gender× Political affiliation	female and unknown	female and Democrats	30.21	44.63	-16.67	186966	3.21×10^{-60}
	female and unknown	female and Republicans	30.21	32.24	-1.27	164811	1.00
	female and unknown	male and unknown	30.21	34.60	-9.06	363926	1.83×10^{-17}
	female and unknown	male and Democrats	30.21	46.30	-19.59	191717	3.12×10^{-83}
	female and unknown	male and Republicans	30.21	38.21	-7.48	175074	1.03×10^{-11}
	female and Democrats	female and Republicans	44.63	32.24	7.17	37048	1.08×10^{-10}
	female and Democrats	male and unknown	44.63	34.60	10.76	236163	7.51×10^{-25}
	female and Democrats	male and Democrats	44.63	46.30	-1.46	63954	1.00
	female and Democrats	male and Republicans	44.63	38.21	5.01	47311	7.89×10^{-5}
	female and Republicans	male and unknown	32.24	34.60	-1.34	214008	1.00
	female and Republicans	male and Democrats	32.24	46.30	-7.86	41799	5.68×10^{-13}
	female and Republicans	male and Republicans	32.24	38.21	-3.62	25156	4.23×10^{-2}
	male and unknown	male and Democrats	34.60	46.30	-13.32	240914	2.58×10^{-38}
	male and unknown	male and Republicans	34.60	38.21	-3.08	224271	2.91×10^{-1}
	male and Democrats	male and Republicans	46.30	38.21	6.24	52062	6.33×10^{-8}
Race/Ethnicity× State density category	White and high	White and low	36.40	34.47	3.39	184653	1.01×10^{-1}
	White and high	Asian and high	36.40	48.87	-12.8	141180	2.46×10^{-35}
	White and high	Asian and low	36.40	42.76	-5.02	124113	7.44×10^{-5}
	White and high	Laxinx and high	36.40	43.71	-10.05	168288	1.40×10^{-21}
	White and high	Laxinx and low	36.40	40.50	-4.74	138321	3.09×10^{-4}
	White and high	Black and high	36.40	26.53	17.28	211350	9.96×10^{-65}
	White and high	Black and low	36.40	24.07	18.85	160433	5.18×10^{-77}
	White and low	Asian and high	34.47	48.87	-13.35	100732	1.76×10^{-38}
	White and low	Asian and low	34.47	42.76	-6.32	83665	3.66×10^{-8}
	White and low	Laxinx and high	34.47	43.71	-11.35	127840	1.07×10^{-27}
	White and low	Laxinx and low	34.47	40.50	-6.68	97873	3.36×10^{-9}
	White and low	Black and high	34.47	26.53	12.28	170902	1.69×10^{-32}
	White and low	Black and low	34.47	24.07	15.02	119985	8.02×10^{-49}
	Asian and high	Asian and low	48.87	42.76	2.59	40192	1.00
	Asian and high	Laxinx and high	48.87	43.71	3.72	84367	2.84×10^{-2}
	Asian and high	Laxinx and low	48.87	40.50	5.19	54400	2.97×10^{-5}
	Asian and high	Black and high	48.87	26.53	20.45	127429	1.25×10^{-90}
	Asian and high	Black and low	48.87	24.07	20.27	76512	6.35×10^{-89}
	Asian and low	Laxinx and high	42.76	43.71	-0.53	67300	1.00
	Asian and low	Laxinx and low	42.76	40.50	1.27	37333	1.00
	Asian and low	Black and high	42.76	26.53	11.18	110362	7.73×10^{-27}
	Asian and low	Black and low	42.76	24.07	13.35	59445	2.02×10^{-38}
	Laxinx and high	Laxinx and low	43.71	40.50	2.61	81508	1.00

Table S8 continued from previous page

Interaction	Group 1	Group 2	Mean mobility difference Group 1	Group 2	t-statistic	DF	Corrected p values
	Laxinx and high	Black and high	43.71	26.53	21.13	154537	7.64×10^{-97}
	Laxinx and high	Black and low	43.71	24.07	21.08	103620	2.75×10^{-96}
	Laxinx and low	Black and high	40.50	26.53	14.13	124570	3.89×10^{-43}
	Laxinx and low	Black and low	40.50	24.07	16.9	73653	8.57×10^{-62}
	Black and high	Black and low	26.53	24.07	3.3	146682	1.41×10^{-1}
Gender× Age× Political affiliation	female and < 30 and unknown	female and < 30 and Democrats	26.93	42.24	-11.77	120200	8.78×10^{-30}
	female and < 30 and unknown	female and < 30 and Republicans	26.93	36.43	-3.24	111090	1.70×10^{-1}
	female and < 30 and unknown	female and ≥ 30 and unknown	26.93	37.64	-14.42	157364	6.01×10^{-45}
	female and < 30 and unknown	female and ≥ 30 and Democrats	26.93	46.05	-18.7	127714	8.29×10^{-76}
	female and < 30 and unknown	female and ≥ 30 and Republicans	26.93	30.77	-2.19	114669	1.00
	female and < 30 and unknown	male and < 30 and unknown	26.93	27.15	-0.38	218661	1.00
	female and < 30 and unknown	male and < 30 and Democrats	26.93	40.13	-9.74	118956	2.96×10^{-20}
	female and < 30 and unknown	male and < 30 and Republicans	26.93	32.19	-2.47	112918	1.00
	female and < 30 and unknown	male and ≥ 30 and unknown	26.93	43.01	-25.6	206213	3.68×10^{-142}
	female and < 30 and unknown	male and ≥ 30 and Democrats	26.93	48.76	-23.29	133709	1.36×10^{-117}
	female and < 30 and unknown	male and ≥ 30 and Republicans	26.93	39.84	-11.25	123104	3.61×10^{-27}
	female and < 30 and Democrats	female and < 30 and Republicans	42.24	36.43	1.62	12977	1.00
	female and < 30 and Democrats	female and ≥ 30 and unknown	42.24	37.64	2.88	59251	5.74×10^{-1}
	female and < 30 and Democrats	female and ≥ 30 and Democrats	42.24	46.05	-2.27	29601	1.00
	female and < 30 and Democrats	female and ≥ 30 and Republicans	42.24	30.77	5.11	16556	4.58×10^{-5}
	female and < 30 and Democrats	male and < 30 and unknown	42.24	27.15	10.33	120548	7.37×10^{-23}
	female and < 30 and Democrats	male and < 30 and Democrats	42.24	40.13	1.06	20843	1.00
	female and < 30 and Democrats	male and < 30 and Republicans	42.24	32.19	3.64	14805	3.88×10^{-2}
	female and < 30 and Democrats	male and ≥ 30 and unknown	42.24	43.01	-0.49	108100	1.00
	female and < 30 and Democrats	male and ≥ 30 and Democrats	42.24	48.76	-3.76	35596	2.46×10^{-2}
	female and < 30 and Democrats	male and ≥ 30 and Republicans	42.24	39.84	1.38	24991	1.00
	female and < 30 and Republicans	female and ≥ 30 and unknown	36.43	37.64	-0.35	50141	1.00
	female and < 30 and Republicans	female and ≥ 30 and Democrats	36.43	46.05	-3.07	20491	3.02×10^{-1}
	female and < 30 and Republicans	female and ≥ 30 and Republicans	36.43	30.77	2.09	7446	1.00
	female and < 30 and Republicans	male and < 30 and unknown	36.43	27.15	2.79	111438	7.64×10^{-1}
	female and < 30 and Republicans	male and < 30 and Democrats	36.43	40.13	-1.14	11733	1.00
	female and < 30 and Republicans	male and < 30 and Republicans	36.43	32.19	1.22	5695	1.00
	female and < 30 and Republicans	male and ≥ 30 and unknown	36.43	43.01	-1.83	98990	1.00
	female and < 30 and Republicans	male and ≥ 30 and Democrats	36.43	48.76	-3.5	26486	6.61×10^{-2}
	female and < 30 and Republicans	male and ≥ 30 and Republicans	36.43	39.84	-1.14	15881	1.00
	female and ≥ 30 and unknown	female and ≥ 30 and Democrats	37.64	46.05	-6.64	66765	4.62×10^{-9}
	female and ≥ 30 and unknown	female and ≥ 30 and Republicans	37.64	30.77	3.28	53720	1.49×10^{-1}
	female and ≥ 30 and unknown	male and < 30 and unknown	37.64	27.15	12.99	157712	2.06×10^{-36}
	female and ≥ 30 and unknown	male and < 30 and Democrats	37.64	40.13	-1.51	58007	1.00
	female and ≥ 30 and unknown	male and < 30 and Republicans	37.64	32.19	2.14	51969	1.00
	female and ≥ 30 and unknown	male and ≥ 30 and unknown	37.64	43.01	-6.21	145264	7.54×10^{-8}
	female and ≥ 30 and unknown	male and ≥ 30 and Democrats	37.64	48.76	-9.35	72760	1.31×10^{-18}
	female and ≥ 30 and unknown	male and ≥ 30 and Republicans	37.64	39.84	-1.57	62155	1.00
	female and ≥ 30 and Democrats	female and ≥ 30 and Republicans	46.05	30.77	7.9	24070	4.09×10^{-13}
	female and ≥ 30 and Democrats	male and < 30 and unknown	46.05	27.15	16.54	128062	3.31×10^{-59}
	female and ≥ 30 and Democrats	male and < 30 and Democrats	46.05	40.13	3.56	28357	5.31×10^{-2}
	female and ≥ 30 and Democrats	male and < 30 and Republicans	46.05	32.19	5.85	22319	7.31×10^{-7}
	female and ≥ 30 and Democrats	male and ≥ 30 and unknown	46.05	43.01	2.48	115614	1.00
	female and ≥ 30 and Democrats	male and ≥ 30 and Democrats	46.05	48.76	-1.93	43110	1.00
	female and ≥ 30 and Democrats	male and ≥ 30 and Republicans	46.05	39.84	4.29	32505	2.60×10^{-3}
	female and ≥ 30 and Republicans	male and < 30 and unknown	30.77	27.15	1.82	115017	1.00
	female and ≥ 30 and Republicans	male and < 30 and Democrats	30.77	40.13	-4.54	15312	8.16×10^{-4}
	female and ≥ 30 and Republicans	male and < 30 and Republicans	30.77	32.19	-0.59	9274	1.00
	female and ≥ 30 and Republicans	male and ≥ 30 and unknown	30.77	43.01	-5.73	102569	1.46×10^{-6}
	female and ≥ 30 and Republicans	male and ≥ 30 and Democrats	30.77	48.76	-8.41	30065	6.14×10^{-15}
	female and ≥ 30 and Republicans	male and ≥ 30 and Republicans	30.77	39.84	-4.83	19460	1.95×10^{-4}
	male and < 30 and unknown	male and < 30 and Democrats	27.15	40.13	-8.5	119304	2.75×10^{-15}
	male and < 30 and unknown	male and < 30 and Republicans	27.15	32.19	-2.09	113266	1.00
	male and < 30 and unknown	male and ≥ 30 and unknown	27.15	43.01	-23.8	206561	6.52×10^{-123}
	male and < 30 and unknown	male and ≥ 30 and Democrats	27.15	48.76	-20.83	134057	4.27×10^{-94}
	male and < 30 and unknown	male and ≥ 30 and Republicans	27.15	39.84	-9.83	123452	1.21×10^{-20}
	male and < 30 and Democrats	male and < 30 and Republicans	40.13	32.19	3.11	13561	2.65×10^{-1}
	male and < 30 and Democrats	male and ≥ 30 and unknown	40.13	43.01	-1.75	106856	1.00
	male and < 30 and Democrats	male and ≥ 30 and Democrats	40.13	48.76	-4.92	34352	1.24×10^{-4}
	male and < 30 and Democrats	male and ≥ 30 and Republicans	40.13	39.84	0.17	23747	1.00
	male and < 30 and Republicans	male and ≥ 30 and unknown	32.19	43.01	-4.17	100818	4.29×10^{-3}
	male and < 30 and Republicans	male and ≥ 30 and Democrats	32.19	48.76	-6.35	28314	3.17×10^{-8}
	male and < 30 and Republicans	male and ≥ 30 and Republicans	32.19	39.84	-3.31	17709	1.36×10^{-1}
	male and ≥ 30 and unknown	male and ≥ 30 and Democrats	43.01	48.76	-5.17	121609	3.44×10^{-5}
	male and ≥ 30 and unknown	male and ≥ 30 and Republicans	43.01	39.84	2.29	111004	1.00
	male and ≥ 30 and Democrats	male and ≥ 30 and Republicans	48.76	39.84	5.91	38500	5.01×10^{-7}

Supplementary Table S8. Post-hoc analysis for significant interactions from the major ANOVA test. T-statistic, DF (degrees of freedom) and corrected P values are shown.

ANOVA Test Results

Interactions	F statistic	DF	P-value	Group	Mean mobility difference	Sample size
Gender×Age	6.16	1.0	1.30×10^{-02}	female and < 30	28.46	122135
				female and ≥ 30	39.27	72279
				male and < 30	28.34	123067
				male and ≥ 30	43.72	135558
Race/Ethnicity×Age	2.73	3.0	4.22×10^{-02}	White and < 30	30.83	103108
				White and ≥ 30	41.74	81546
				Asian and < 30	36.50	20754
				Asian and ≥ 30	58.45	19439
				Latinx and < 30	34.57	24853
				Latinx and ≥ 30	46.26	56656
				Black and < 30	22.47	96487
				Black and ≥ 30	31.97	50196
Gender×Political affiliation	3.62	2.0	2.68×10^{-02}	female and unknown	30.21	157365
				female and Democrats	44.63	29602
				female and Republicans	32.24	7447
				male and unknown	34.60	206562
				male and Democrats	46.30	34353
				male and Republicans	38.21	17710
Race/Ethnicity×State population density category	4.40	3.0	4.23×10^{-03}	White and high	36.40	112551
				White and low	34.47	72103
				Asian and high	48.87	28630
				Asian and low	42.76	11563
				Latinx and high	43.71	55738
				Latinx and low	40.50	25771
				Black and high	26.53	98800
				Black and low	24.07	47883
Gender×Age×Political affiliation	3.66	2.0	2.58×10^{-02}	female and < 30 and unknown	26.93	109157
				female and < 30 and Democrats	42.24	11044
				female and < 30 and Republicans	36.43	1934
				female and ≥ 30 and unknown	37.64	48208
				female and ≥ 30 and Democrats	46.05	18558
				female and ≥ 30 and Republicans	30.77	5513
				male and < 30 and unknown	27.15	109505
				male and < 30 and Democrats	40.13	9800
				male and < 30 and Republicans	32.19	3762
				male and ≥ 30 and unknown	43.01	97057
				male and ≥ 30 and Democrats	48.76	24553
				male and ≥ 30 and Republicans	39.84	13948

Supplementary Table S9. Summary statistics of the major ANOVA tests and mean mobility differences of significant interactions. Independent variables include age, gender, race/ethnicity, political affiliation and state population density. F statistic, DF (degrees of freedom), and p value shown for each interaction.

Variable/Interaction	F statistic	DF	P-value	Group	Mean mobility difference	Sample size
Gender	4.26	1.0	3.89×10^{-02}	female	32.48	194414
				male	36.40	258625
Race/Ethnicity	9.57	3.0	2.57×10^{-06}	White	35.65	184654
				Asian	47.11	40193
				Latinx	42.70	81509
				Black	25.72	146683
COVID-19 hashtags	29.71	1.0	5.02×10^{-08}	False	31.27	295180
				True	41.17	157859
State population density category	5.40	1.0	2.02×10^{-02}	high	35.69	295719
				low	32.90	157320
Gender×Age	5.81	1.0	1.59×10^{-02}	female and < 30	28.46	122135
				female and ≥ 30	39.27	72279
				male and < 30	28.34	123067
				male and ≥ 30	43.72	135558
Race/Ethnicity×COVID-19 hashtags	5.11	3.0	1.56×10^{-03}	White and False	33.05	125249
				White and True	41.12	59405
				Asian and False	41.08	24655
				Asian and True	56.70	15538
				Latinx and False	37.58	45178
				Latinx and True	49.06	36331
				Black and False	23.78	100098
				Black and True	29.90	46585
Age×COVID-19 hashtags	17.42	1.0	2.99×10^{-05}	< 30 and False	26.99	179388
				< 30 and True	32.24	65814
				≥ 30 and False	37.90	115792
				≥ 30 and True	47.56	92045
Race/Ethnicity×State population density category	3.05	3.0	2.73×10^{-02}	White and high	36.40	112551
				White and low	34.47	72103
				Asian and high	48.87	28630
				Asian and low	42.76	11563
				Latinx and high	43.71	55738
				Latinx and low	40.50	25771
				Black and high	26.53	98800
				Black and low	24.07	47883
Race/Ethnicity×Age×COVID-19 hashtags	4.34	3.0	4.62×10^{-03}	White and < 30 and False	29.72	78810
				White and < 30 and True	34.43	24298
				White and ≥ 30 and False	38.70	46439
				White and ≥ 30 and True	45.76	35107
				Asian and < 30 and False	35.00	13917
				Asian and < 30 and True	39.54	6837
				Asian and ≥ 30 and False	48.95	10738
				Asian and ≥ 30 and True	70.18	8701
				Latinx and < 30 and False	31.45	15520
				Latinx and < 30 and True	39.77	9333
				Latinx and ≥ 30 and False	40.79	29658
				Latinx and ≥ 30 and True	52.27	26998
				Black and < 30 and False	21.43	71141
				Black and < 30 and True	25.41	25346
				Black and ≥ 30 and False	29.56	28957
				Black and ≥ 30 and True	35.26	21239

Supplementary Table S10. Summary statistics of the validation ANOVA tests and mean mobility differences of significant variables and interactions. Independent variables include age, gender, race/ethnicity, state population density and indicator of whether a user mentions COVID19 hashtags. F statistic, DF (degrees of freedom), and p value shown for each variable and interaction.

Variable/Interaction	F statistic	DF	P-value	Group	Mean mobility difference	Sample size
Race/Ethnicity	26.14	3.0	6.73×10^{-17}	White	35.65	184654
				Asian	47.11	40193
				Latinx	42.70	81509
				Black	25.72	146683
Age	18.77	1.0	1.47×10^{-05}	< 30	28.40	245202
				≥ 30	42.18	207837
State population density category	9.67	1.0	1.88×10^{-03}	high	35.69	295719
				low	32.90	157320
Gender×Age	5.00	1.0	2.53×10^{-02}	female and < 30	28.46	122135
				female and ≥ 30	39.27	72279
				male and < 30	28.34	123067
				male and ≥ 30	43.72	135558
Race/Ethnicity×State population density category	4.21	3.0	5.50×10^{-03}	White and high	36.40	112551
				White and low	34.47	72103
				Asian and high	48.87	28630
				Asian and low	42.76	11563
				Latinx and high	43.71	55738
				Latinx and low	40.50	25771
				Black and high	26.53	98800
				Black and low	24.07	47883

Supplementary Table S11. Summary statistics of the validation ANOVA tests and mean mobility differences of significant variables and interactions. Independent variables include age, gender, race/ethnicity, state population density and indicator of whether a user mentions social-distancing hashtags. F statistic, DF (degrees of freedom), and p value shown for each variable and interaction.

Variable/Interaction	F statistic	DF	P-value	Group	Mean mobility difference	Sample size
Race/Ethnicity	31.89	3.0	1.33×10^{-20}	White	35.65	184654
				Asian	47.11	40193
				Latinx	42.70	81509
				Black	25.72	146683
Age	41.09	1.0	1.45×10^{-10}	< 30	28.40	245202
				≥ 30	42.18	207837
Follow realDonaldTrump	9.59	1.0	1.96×10^{-03}	False	34.60	348393
				True	35.12	104646
State population density category	9.17	1.0	2.47×10^{-03}	high	35.69	295719
				low	32.90	157320
Race/Ethnicity×Age	4.61	3.0	3.17×10^{-03}	White and < 30	30.83	103108
				White and ≥ 30	41.74	81546
				Asian and < 30	36.50	20754
				Asian and ≥ 30	58.45	19439
				Latinx and < 30	34.57	24853
				Latinx and ≥ 30	46.26	56656
				Black and < 30	22.47	96487
				Black and ≥ 30	31.97	50196
Gender×Follow realDonaldTrump	3.86	1.0	4.95×10^{-02}	female and False	32.71	158140
				female and True	31.49	36274
				male and False	36.17	190253
				male and True	37.05	68372
Race/Ethnicity×State population density category	3.57	3.0	1.35×10^{-02}	White and high	36.40	112551
				White and low	34.47	72103
				Asian and high	48.87	28630
				Asian and low	42.76	11563
				Latinx and high	43.71	55738
				Latinx and low	40.50	25771
				Black and high	26.53	98800
				Black and low	24.07	47883
Gender×Race/Ethnicity×Age	2.76	3.0	4.06×10^{-02}	female and White and < 30	30.14	61838
				female and White and ≥ 30	38.47	26583
				female and Asian and < 30	36.56	7879
				female and Asian and ≥ 30	53.07	4675
				female and Latinx and < 30	35.46	13486
				female and Latinx and ≥ 30	42.59	26250
				female and Black and < 30	21.74	38932
				female and Black and ≥ 30	30.46	14771
				male and White and < 30	31.86	41270
				male and White and ≥ 30	43.32	54963
				male and Asian and < 30	36.46	12875
				male and Asian and ≥ 30	60.15	14764
				male and Latinx and < 30	33.53	11367
				male and Latinx and ≥ 30	49.43	30406
				male and Black and < 30	22.97	57555
				male and Black and ≥ 30	32.60	35425

Supplementary Table S12. Summary statistics of the ANOVA tests and mean mobility differences of significant variables and interactions. Independent variables include age, gender, race/ethnicity, state population density and indicator of whether a user follows former U.S. president Trump. F statistic, DF (degrees of freedom), and p value shown for each variable and interaction.

Variable	F statistic	DF	P-value	Group	Mean mobility difference	Sample size
Gender	58.38	1.0	2.16×10^{-14}	female	32.48	194414
				male	36.40	258625
Race/Ethnicity	262.22	3.0	4.78×10^{-170}	White	35.65	184654
				Asian	47.11	40193
				Latinx	42.70	81509
				Black	25.72	146683
Age	405.62	1.0	3.61×10^{-90}	< 30	28.40	245202
				≥ 30	42.18	207837
Political affiliation	67.22	2.0	6.50×10^{-30}	unknown	32.70	363927
				Democrats	45.53	63955
				Republicans	36.44	25157
COVID-19 hashtags	108.02	1.0	2.68×10^{-25}	False	31.27	295180
				True	41.17	157859
Social-distancing hashtags	48.64	1.0	3.08×10^{-12}	False	32.77	360463
				True	42.32	92576
State population density category	12.97	1.0	3.16×10^{-04}	high	35.69	295719
				low	32.90	157320
Follow realDonaldTrump	36.12	1.0	1.85×10^{-09}	False	34.60	348393
				True	35.12	104646

Supplementary Table S13. Summary statistics of the one-way ANOVA test and mean mobility differences of significant variables. Independent variables include age, gender, race/ethnicity, state population density, and indicators of whether a user mentions COVID19 or social-distancing hashtags and when a user follows the former U.S. president Trump. F statistic, DF (degrees of freedom), and p value shown for each variable.

Variable/Intersection	F	DF	P-value	Group	MMD	Sample size
Gender	6.8	1	9.13×10^{-03}	female	32.48	194414
				male	36.4	258625
Race/Ethnicity	24.5	3	7.60×10^{-16}	Asian	47.11	40193
				Black	25.72	146683
				White	35.65	184654
				Latinx	42.7	81509
Age	22.85	1	1.75×10^{-06}	< 30	28.4	245202
				≥ 30	42.18	207837
Political affiliation	8.05	2	3.18×10^{-4}	unknown	32.7	363927
				Republicans	36.44	25157
				Democrats	45.53	63955
State population density category	10.5	1	1.19×10^{-3}	low	32.9	157320
				high	35.69	295719
Gender × Race/Ethnicity	1.27	3	2.84×10^{-01}	female and Asian	42.71	12554
				female and Black	24.14	53703
				female and White	32.64	88421
				female and Latinx	40.17	39736
				male and Asian	49.12	27639
				male and Black	26.64	92980
				male and White	38.41	96233
				male and Latinx	45.1	41773
Gender × Age	6.16	1	1.30×10^{-02}	female and < 30	28.46	122135
				female and ≥ 30	39.27	72279
				male and < 30	28.34	123067
				male and ≥ 30	43.72	135558
Race/Ethnicity × Age	2.73	3	4.22×10^{-02}	Asian and < 30	36.5	20754
				Asian and ≥ 30	58.45	19439
				Black and < 30	22.47	96487
				Black and ≥ 30	31.97	50196
				White and < 30	30.83	103108
				White and ≥ 30	41.74	81546
				Latinx and < 30	34.57	24853
				Latinx and ≥ 30	46.26	56656
Gender × Political affiliation	3.62	2	2.68×10^{-02}	female and unknown	30.21	157365
				female and Republicans	32.24	7447
				female and Democrats	44.63	29602
				male and unknown	34.6	206562
				male and Republicans	38.21	17710
				male and Democrats	46.3	34353
Race/Ethnicity × Political affiliation	2.01	6	6.07×10^{-02}	Asian and unknown	44.77	33553
				Asian and Republicans	47.1	1302
				Asian and Democrats	61.86	5338
				Black and unknown	24.23	125289
				Black and Republicans	29.49	5118
				Black and Democrats	36.02	16276
				White and unknown	33.75	147655
				White and Republicans	38	12416
				White and Democrats	45.86	24583
				Latinx and unknown	41.44	57430
				Latinx and Republicans	36.82	6321
				Latinx and Democrats	48.87	17758
Age × Political affiliation	2.46	2	8.56×10^{-02}	< 30 and unknown	27.04	218662
				< 30 and Republicans	33.63	5696
				< 30 and Democrats	41.25	20844
				≥ 30 and unknown	41.22	145265
				≥ 30 and Republicans	37.27	19461
				≥ 30 and Democrats	47.59	43111
Gender × State population density category	1.62	1	2.03×10^{-01}	female and low	31.36	69869
				female and high	33.12	124545
				male and low	34.13	87451
				male and high	37.56	171174
Race/Ethnicity × State population density category	4.4	3	4.23×10^{-03}	Asian and low	42.76	11563
				Asian and high	48.87	28630
				Black and low	24.07	47883
				Black and high	26.53	98800
				White and low	34.47	72103
				White and high	36.4	112551
				Latinx and low	40.5	25771
				Latinx and high	43.71	55738
Age × State population density category	3.03	1	8.20×10^{-02}	< 30 and low	27.06	89892
				< 30 and high	29.18	155310
				≥ 30 and low	40.69	67428
				≥ 30 and high	42.89	140409
Political affiliation × State population density category	0.4	2	6.71×10^{-01}	unknown and low	31.08	129159
				unknown and high	33.59	234768
				Republicans and low	36.23	9973
				Republicans and high	36.58	15184
				Democrats and low	43.99	18188
				Democrats and high	46.14	45767

Table S14 continued from previous page

Variable/Intersection	F	DF	P-value	Group	MMD	Sample size
Gender × Race/Ethnicity × Age	1.91	3	1.26×10^{-01}	female and Asian and < 30	36.56	7879
				female and Asian and ≥ 30	53.07	4675
				female and Black and < 30	21.74	38932
				female and Black and ≥ 30	30.46	14771
				female and White and < 30	30.14	61838
				female and White and ≥ 30	38.47	26583
				female and Latinx and < 30	35.46	13486
				female and Latinx and ≥ 30	42.59	26250
				male and Asian and < 30	36.46	12875
				male and Asian and ≥ 30	60.15	14764
				male and Black and < 30	22.97	57555
				male and Black and ≥ 30	32.6	35425
				male and White and < 30	31.86	41270
				male and White and ≥ 30	43.32	54963
				male and Latinx and < 30	33.53	11367
				male and Latinx and ≥ 30	49.43	30406
Gender × Race/Ethnicity × Political affiliation	0.94	6	4.68×10^{-01}	female and Asian and unknown	39.44	10617
				female and Asian and Republicans	45.92	256
				female and Asian and Democrats	62.85	1681
				female and Black and unknown	22.38	45217
				female and Black and Republicans	23.1	1365
				female and Black and Democrats	35.52	7121
				female and White and unknown	30.57	73629
				female and White and Republicans	36.04	3308
				female and White and Democrats	44.96	11484
				female and Latinx and unknown	38.43	27902
				female and Latinx and Republicans	30.81	2518
				female and Latinx and Democrats	47.91	9316
				male and Asian and unknown	47.23	22936
				male and Asian and Republicans	47.39	1046
				male and Asian and Democrats	61.4	3657
				male and Black and unknown	25.28	80072
				male and Black and Republicans	31.82	3753
				male and Black and Democrats	36.4	9155
				male and White and unknown	36.91	74026
				male and White and Republicans	38.71	9108
				male and White and Democrats	46.66	13099
				male and Latinx and unknown	44.28	29528
				male and Latinx and Republicans	40.81	3803
				male and Latinx and Democrats	49.92	8442
Gender × Age × Political affiliation	3.66	2	2.58×10^{-02}	female and < 30 and unknown	26.93	109157
				female and < 30 and Republicans	36.43	1934
				female and < 30 and Democrats	42.24	11044
				female and ≥ 30 and unknown	37.64	48208
				female and ≥ 30 and Republicans	30.77	5513
				female and ≥ 30 and Democrats	46.05	18558
				male and < 30 and unknown	27.15	109505
				male and < 30 and Republicans	32.19	3762
				male and < 30 and Democrats	40.13	9800
				male and ≥ 30 and unknown	43.01	97057
				male and ≥ 30 and Republicans	39.84	13948
				male and ≥ 30 and Democrats	48.76	24553
Race/Ethnicity × Age × Political affiliation	1.13	6	3.43×10^{-01}	Asian and < 30 and unknown	35.23	18484
				Asian and < 30 and Republicans	39.88	372
				Asian and < 30 and Democrats	48.13	1898
				Asian and ≥ 30 and unknown	56.47	15069
				Asian and ≥ 30 and Republicans	49.99	930
				Asian and ≥ 30 and Democrats	69.43	3440
				Black and < 30 and unknown	21.65	88979
				Black and < 30 and Republicans	30.78	1179
				Black and < 30 and Democrats	32.45	6329
				Black and ≥ 30 and unknown	30.55	36310
				Black and ≥ 30 and Republicans	29.11	3939
				Black and ≥ 30 and Democrats	38.29	9947
				White and < 30 and unknown	29.4	91103
				White and < 30 and Republicans	34.26	3242
				White and < 30 and Democrats	44.48	8763
				White and ≥ 30 and unknown	40.76	56552
				White and ≥ 30 and Republicans	39.32	9174
				White and ≥ 30 and Democrats	46.63	15820
				Latinx and < 30 and unknown	32.67	20096
				Latinx and < 30 and Republicans	32.51	903
				Latinx and < 30 and Democrats	44.97	3854
				Latinx and ≥ 30 and unknown	46.15	37334
				Latinx and ≥ 30 and Republicans	37.54	5418
				Latinx and ≥ 30 and Democrats	49.95	13904
Gender × Race/Ethnicity × State population density category	1.33	3	2.62×10^{-01}	female and Asian and low	35.1	3514
				female and Asian and high	45.67	9040
				female and Black and low	23.21	17574

Table S14 continued from previous page

Variable/Intersection	F	DF	P-value	Group	MMD	Sample size
				female and Black and high	24.59	36129
				female and White and low	31.86	35577
				female and White and high	33.18	52844
				female and Latinx and low	39.85	13204
				female and Latinx and high	40.33	26532
				male and Asian and low	46.11	8049
				male and Asian and high	50.35	19590
				male and Black and low	24.57	30309
				male and Black and high	27.64	62671
				male and White and low	37.01	36526
				male and White and high	39.26	59707
				male and Latinx and low	41.19	12567
				male and Latinx and high	46.79	29206
Gender × Age × State population density category	0	1	9.73×10^{-01}	female and <30 and low	27.15	46067
				female and <30 and high	29.26	76068
				female and ≥ 30 and low	39.5	23802
				female and ≥ 30 and high	39.16	48477
				male and <30 and low	26.96	43825
				male and <30 and high	29.1	79242
				male and ≥ 30 and low	41.34	43626
				male and ≥ 30 and high	44.85	91932
Race/Ethnicity × Age × State population density category	1.94	3	1.21×10^{-01}	Asian and <30 and low	35.11	6113
				Asian and <30 and high	37.08	14641
				Asian and ≥ 30 and low	51.35	5450
				Asian and ≥ 30 and high	61.22	13989
				Black and <30 and low	21.16	32896
				Black and <30 and high	23.15	63591
				Black and ≥ 30 and low	30.45	14987
				Black and ≥ 30 and high	32.62	35209
				White and <30 and low	29.51	42478
				White and <30 and high	31.76	60630
				White and ≥ 30 and low	41.58	29625
				White and ≥ 30 and high	41.83	51921
				Latinx and <30 and low	31.9	8405
				Latinx and <30 and high	35.94	16448
				Latinx and ≥ 30 and low	44.66	17366
				Latinx and ≥ 30 and high	46.97	39290
Gender × Political affiliation × State population density category	0.54	2	5.83×10^{-01}	female and unknown and low	29.25	58377
				female and unknown and high	30.77	98988
				female and Republicans and low	35.35	2968
				female and Republicans and high	30.17	4479
				female and Democrats and low	44.38	8524
				female and Democrats and high	44.73	21078
				male and unknown and low	32.59	70782
				male and unknown and high	35.65	135780
				male and Republicans and low	36.6	7005
				male and Republicans and high	39.27	10705
				male and Democrats and low	43.64	9664
				male and Democrats and high	47.34	24689
Race/Ethnicity × Political affiliation × State population density category	0.52	6	7.95×10^{-01}	Asian and unknown and low	40.67	9818
				Asian and unknown and high	46.46	23735
				Asian and Republicans and low	42.63	437
				Asian and Republicans and high	49.37	865
				Asian and Democrats and low	58.51	1308
				Asian and Democrats and high	62.94	4030
				Black and unknown and low	22.69	41651
				Black and unknown and high	25	83638
				Black and Republicans and low	31.38	1870
				Black and Republicans and high	28.41	3248
				Black and Democrats and low	34.12	4362
				Black and Democrats and high	36.71	11914
				White and unknown and low	32.89	58946
				White and unknown and high	34.32	88709
				White and Republicans and low	37.79	5323
				White and Republicans and high	38.15	7093
				White and Democrats and low	44.09	7834
				White and Democrats and high	46.69	16749
				Latinx and unknown and low	39.04	18744
				Latinx and unknown and high	42.6	38686
				Latinx and Republicans and low	35.36	2343
				Latinx and Republicans and high	37.69	3978
				Latinx and Democrats and low	48.94	4684
				Latinx and Democrats and high	48.84	13074
Age × Political affiliation × State population density category	0.82	2	4.39×10^{-01}	<30 and unknown and low	25.9	81332
				<30 and unknown and high	27.71	137330
				<30 and Republicans and low	34.45	2350
				<30 and Republicans and high	33.05	3346
				<30 and Democrats and low	39.38	6210
				<30 and Democrats and high	42.04	14634

Table S14 continued from previous page

Variable/Intersection	F	DF	P-value	Group	MMD	Sample size
				$\geq$ 30 and unknown and low	39.89	47827
				$\geq$ 30 and unknown and high	41.88	97438
				$\geq$ 30 and Republicans and low	36.78	7623
				$\geq$ 30 and Republicans and high	37.58	11838
				$\geq$ 30 and Democrats and low	46.37	11978
				$\geq$ 30 and Democrats and high	48.06	31133

Supplementary Table S14. Supplement of Table 10. Summary statistics of the major ANOVA test and mean mobility differences (MMD) of interactions up to 3 way. Independent variables include age, gender, race/ethnicity, political affiliation and state population density. F (F statistics), DF (degrees of freedom), and p value shown for each variable/interaction.

Mobility Reduction Distribution

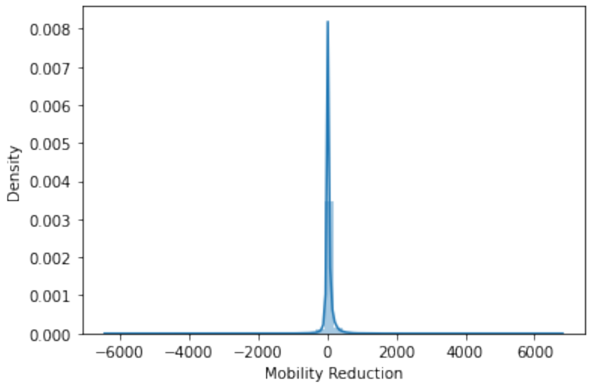

(a) All users

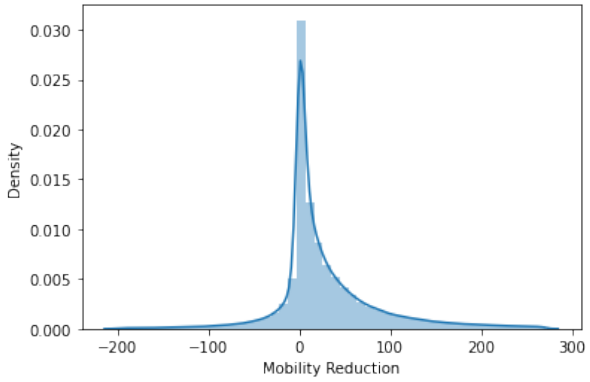

(b) Users in 90% confidence interval

Supplementary Figure S1. Original Mobility reduction distribution.

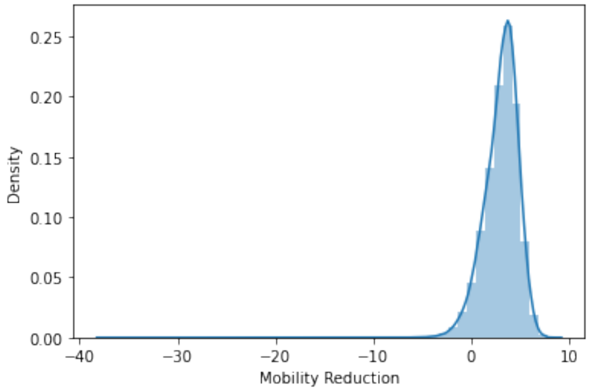

(a) Users with mobility reductions over 0

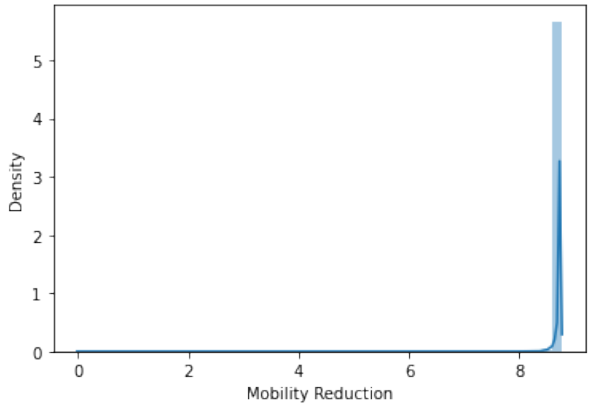

(b) Users with mobility reductions below 0

Supplementary Figure S2. The log-transformed mobility reduction distribution.

Regression Analysis

	MR I	MR II	MR III	MR IIII	MR IIIII
Age [>30]	8.81***<br>(0.46)				
Gender [female]	-5.70***<br>(0.44)	-6.29***<br>(0.44)	-6.20***<br>(0.44)	-6.29***<br>(0.44)	-6.20***<br>(0.44)
Political affiliation [unknown]	-4.60***<br>(0.62)	-4.60***<br>(0.62)	-4.20***<br>(0.62)	-4.38***<br>(0.62)	-3.96***<br>(0.62)
Political affiliation [Republican]	-8.46***<br>(1.07)	-9.46***<br>(1.07)	-8.41***<br>(1.07)	-9.15***<br>(1.07)	-8.08***<br>(1.07)
Race/Ethnicity [Asian]	23.92***<br>(0.79)				
Race/Ethnicity [Black]	-8.22***<br>(0.51)				
Race/Ethnicity [Latinx]	11.39***<br>(0.62)				
Intercept	62.25***<br>(0.77)	58.94***<br>(0.70)	55.73***<br>(0.75)	58.29***<br>(0.70)	55.02***<br>(0.75)
R-squared	0.01<br>0.01	0.01<br>0.01	0.01<br>0.01	0.01<br>0.01	0.01<br>0.01
Age trust level		7.99***<br>(0.46)	8.19***<br>(0.46)	7.93***<br>(0.46)	8.13***<br>(0.46)
Race/Ethnicity trust level		23.98***<br>(0.57)	23.35***<br>(0.57)	23.95***<br>(0.57)	23.31***<br>(0.57)
State COVID-19 count	10.99***<br>(0.88)		10.76***<br>(0.88)		10.89***<br>(0.88)
State population density				15.93***<br>(2.29)	16.48***<br>(2.29)

Supplementary Table S15. Linear regression result of users with Mobility Reduction (MR) over 0. Standard errors are in parentheses. * p<.1, ** p<.05, ***p<.01

	MR I	MR II	MR III	MR IIII	MR IIIII
Age [>30]	0.07***<br>(0.01)				
Gender [female]	-0.08***<br>(0.01)	-0.06***<br>(0.01)	-0.07***<br>(0.01)	-0.06***<br>(0.01)	-0.07***<br>(0.01)
Political affiliation [unknown]	-0.05***<br>(0.01)	-0.04***<br>(0.01)	-0.05***<br>(0.01)	-0.04***<br>(0.01)	-0.05***<br>(0.01)
Political affiliation [Republican]	-0.04***<br>(0.01)	-0.01<br>(0.01)	-0.03**<br>(0.01)	-0.01<br>(0.01)	-0.03**<br>(0.01)
Race/Ethnicity [Asian]	0.02***<br>(0.01)				
Race/Ethnicity [Black]	-0.22***<br>(0.01)				
Race/Ethnicity [Latinx]	-0.06***<br>(0.01)				
Intercept	3.38***<br>(0.01)	3.21***<br>(0.01)	3.25***<br>(0.01)	3.21***<br>(0.01)	3.26***<br>(0.01)
R-squared	0.01<br>0.01	0.00<br>0.00	0.00<br>0.00	0.00<br>0.00	0.00<br>0.00
Age trust level		0.08***<br>(0.01)	0.08***<br>(0.01)	0.08***<br>(0.01)	0.08***<br>(0.01)
Race/Ethnicity trust level		0.12***<br>(0.01)	0.13***<br>(0.01)	0.12***<br>(0.01)	0.13***<br>(0.01)
State COVID-19 count	-0.13***<br>(0.01)		-0.16***<br>(0.01)		-0.16***<br>(0.01)
State population density				-0.11***<br>(0.03)	-0.12***<br>(0.03)

Supplementary Table S16. Linear regression result of users with Mobility Reduction (MR) over 0. Mobility reduction is log-transformed using $log(x+1)$. Standard errors are in parentheses. * p<.1, ** p<.05, ***p<.01

	MR I	MR II	MR III	MR IIII	MR IIIII
Age [>30]	0.27***<br>(0.01)				
Gender [female]	0.06***<br>(0.01)	0.07***<br>(0.01)	0.07***<br>(0.01)	0.07***<br>(0.01)	0.07***<br>(0.01)
Political affiliation [unknown]	-0.28***<br>(0.01)	-0.29***<br>(0.01)	-0.28***<br>(0.01)	-0.28***<br>(0.01)	-0.28***<br>(0.01)
Political affiliation [Republican]	-0.29***<br>(0.02)	-0.29***<br>(0.02)	-0.27***<br>(0.02)	-0.28***<br>(0.02)	-0.27***<br>(0.02)
Race/Ethnicity [Asian]	0.09***<br>(0.01)				
Race/Ethnicity [Black]	-0.19***<br>(0.01)				
Race/Ethnicity [Latinx]	-0.01<br>(0.01)				
Intercept	1.45***<br>(0.01)	1.38***<br>(0.01)	1.33***<br>(0.01)	1.37***<br>(0.01)	1.32***<br>(0.01)
Age trust level		0.26***<br>(0.01)	0.27***<br>(0.01)	0.26***<br>(0.01)	0.27***<br>(0.01)
Race/Ethnicity trust level		0.19***<br>(0.01)	0.17***<br>(0.01)	0.19***<br>(0.01)	0.17***<br>(0.01)
State COVID-19 count	0.21***<br>(0.02)		0.19***<br>(0.02)		0.19***<br>(0.02)
State population density				0.32***<br>(0.05)	0.32***<br>(0.05)

Supplementary Table S17. Logistic regression result comparing users who have Mobility Reduction (MR) over 0 to those who have reduction of 0 or less. Standard errors are in parentheses. * p<.1, ** p<.05, ***p<.01
